# Methyl 2-(2-hydroxy­benzyl­ideneamino)-4,5,6,7-tetra­hydro-1-benzothio­phene-3-carboxyl­ate

**DOI:** 10.1107/S1600536808010489

**Published:** 2008-04-18

**Authors:** Mehmet Akkurt, Selvi Karaca, Abdullah Mohamed Asiri, Orhan Büyükgüngör

**Affiliations:** a Department of Physics, Faculty of Arts and Sciences, Erciyes University, 38039 Kayseri, Turkey; b Chemistry Department, Faculty of Science, King Abdul-Aziz University, PO Box 80203, Jeddah 21589, Saudi Arabia; c Department of Physics, Faculty of Arts and Sciences, Ondokuz Mayıs University, 55139 Samsun, Turkey

## Abstract

In the title compound, C_17_H_17_NO_3_S, the cyclohexene ring is essentially planar, with a maximum deviation of 0.006 (1) Å. The cyclo­hexene ring adopts a half-chair conformation. The dihedral angle between the thio­phene and benzene rings is 29.7 (1)°. The mol­ecular structure exhibits intra­molecular O—H⋯O, O—H⋯N and C—H⋯S hydrogen bonds, which generate one *S*(5) and two *S*(6) motifs. There is also a C—H⋯π inter­action between the cyclo­hexene ring system and the π-system of the benzene ring.

## Related literature

For related literature, see: Akkurt *et al.* (2008 ▶); Allen *et al.* (1987 ▶); Asiri & Badahdah (2007 ▶); Bernstein *et al.* (1995 ▶); Cremer & Pople (1975 ▶); Etter (1990 ▶).
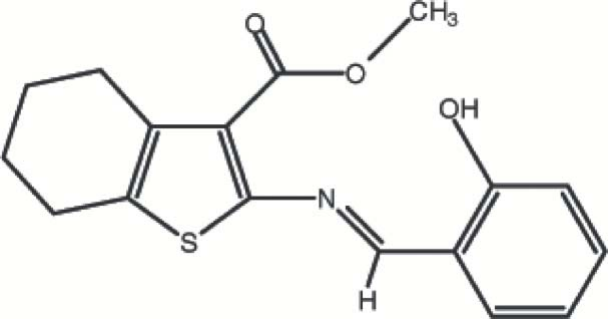


## Experimental

### 

#### Crystal data

C_17_H_17_NO_3_S
*M* *_r_* = 315.39
Monoclinic, 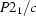

*a* = 7.6107 (4) Å
*b* = 21.2154 (9) Å
*c* = 11.1827 (7) Å
β = 123.342 (4)°
*V* = 1508.41 (16) Å^3^
*Z* = 4
Mo *K*α radiation
μ = 0.23 mm^−1^
*T* = 293 (2) K
0.60 × 0.47 × 0.23 mm

#### Data collection

Stoe IPDS2 diffractometer
Absorption correction: integration (**X-RED32**; Stoe & Cie, 2002 ▶) *T*_min_ = 0.876, *T*_max_ = 0.950
8036 measured reflections
3086 independent reflections
2361 reflections with *I* > 2σ(*I*)
*R*_int_ = 0.078

#### Refinement

*R*[*F* ^2^ > 2σ(*F* ^2^)] = 0.045
*wR*(*F* ^2^) = 0.121
*S* = 0.99
3086 reflections
200 parameters
H-atom parameters constrained
Δρ_max_ = 0.34 e Å^−3^
Δρ_min_ = −0.41 e Å^−3^

### 

Data collection: *X-AREA* (Stoe & Cie, 2002 ▶); cell refinement: *X-AREA*; data reduction: *X-RED32* (Stoe & Cie, 2002 ▶); program(s) used to solve structure: *SHELXS97* (Sheldrick, 2008 ▶); program(s) used to refine structure: *SHELXL97* (Sheldrick, 2008 ▶); molecular graphics: *ORTEP-3 for Windows* (Farrugia, 1997 ▶); software used to prepare material for publication: *WinGX* (Farrugia, 1999 ▶).

## Supplementary Material

Crystal structure: contains datablocks global, I. DOI: 10.1107/S1600536808010489/is2289sup1.cif

Structure factors: contains datablocks I. DOI: 10.1107/S1600536808010489/is2289Isup2.hkl

Additional supplementary materials:  crystallographic information; 3D view; checkCIF report

## Figures and Tables

**Table 1:** Hydrogen-bond geometry (Å, °)

*D*—H⋯*A*	*D*—H	H⋯*A*	*D*⋯*A*	*D*—H⋯*A*
O1—H1⋯O2	0.82	2.50	3.102 (2)	132
O1—H1⋯N1	0.82	1.88	2.607 (2)	146
C7—H7⋯S1	0.93	2.69	3.0725 (19)	105
C15—H15*A*⋯*Cg*^i^	0.97	2.92	3.782 (3)	150

Symmetry code: (i) 
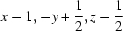
. *Cg* is the centroid of the benzene ring.

**Fig. 1.**
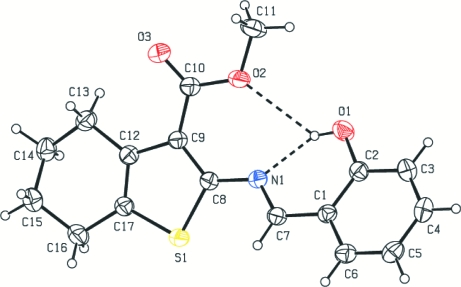
An ORTEP-3 view of the title compound (I), with the atom-numbering scheme, intramolecular H-bonds and 30% probability displacement ellipsoids.

**Fig. 2.**
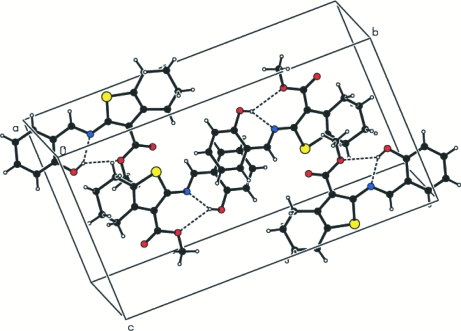
A view of the packing of (I) in the unit cell.

## supplementary crystallographic information

### Comment

In a previous paper, we reported the structure of 4-[(2-hydroxy-1-naphthyl)methylideneamino]benzoic acid (Akkurt *et al.*, 2008). The present work is part of an ongoing investigation in the development of anil derivatives. Here, we report the structure of the title compound 2-[(2-hydroxybenzylidene)amino]-3-methoxycarbonyl-3,4,5,6- tetrahydrobenzo[*d*]thiophene, (I).

In the title compound (I) (Fig. 1), all bond lengths and angles are in normal range (Allen *et al.*, 1987). The thiophene ring is essentially planar, with maximum deviations of 0.006 (1) Å for S1 and 0.006 (3) Å for C17. The cyclohexene ring adopts a half-chair conformation, with the puckering parameters Q_T_ = 0.502 (2) Å, θ = 52.1 (2)° and φ = 151.4 (3)° (Cremer & Pople, 1975). The dihedral angle between the thiophene ring and the benzene ring is 29.7 (1)°.

In the molecular structure, the intramolecular O1—H1···O2, and O1—H1···N1 hydrogen bonds form two pseudo-six membered rings [S(6) motifs (Bernstein *et al.*, 1995; Etter, 1990)] and C7—H7···S1 forms a pseudo five membered ring [S(5) motif], thus locking the molecular conformation and eliminating flexibility (Fig. 1 and Table 1). There are also interactions of the C—H···π type [C15—H15A···π (-1 + *x*, 1/2 - *y*, -1/2 + *z*), H15···π = 2.91 Å, C15···π = 3.782 (3) Å] observed between the cyclohexene ring system and the π system of the benzene ring. A view of the packing of the title compound in the unit cell is shown in Fig. 2.

### Experimental

A solution of 2-amino-3-methoxycarbonyl-3,4,5,6-tetrahydrobenzo[*d*]thieophene (2.0 g, 6.35 mmol) in pure ethanol was heated to its boiling temperature, and then 2-hydroxynaphthaldehyde (0.77 g, 6.35 mmol) dissolved in hot ethanol was added to the amine solution and the resulting mixture was then refluxed for 5 h. Cooling the mixture, filtering the precipitates and recrystalization from ethanol gave the pure product (Asiri & Badahdah, 2007). IR (KBr) ν (cm^-1^); 1697 (C═O), 1604 (C═N), 1443 (C═C), 1323 (C—O) and 1136 (C—N) [yield 98%, m.p. 417 K].

### Refinement

The H atoms were positioned geometrically and allowed to ride on their parent atoms, with the C—H distances in the range of 0.93 - 0.97 Å and O—H = 0.82 Å, and *U*_iso_(H) = 1.2*U*_eq_ (C_aromatic_, C_methylene_) and 1.5*U*_eq_ (C_methyl_, O).

### Crystal data

**Table:**

C_17_H_17_NO_3_S	*F*_000_ = 664
*M**_r_* = 315.39	*D*_x_ = 1.389 Mg m^−^^3^
Monoclinic, *P*2_1_/*c*	Mo *K*α radiation λ = 0.71073 Å
Hall symbol: -P 2ybc	Cell parameters from 10365 reflections
*a* = 7.6107 (4) Å	θ = 1.9–27.2º
*b* = 21.2154 (9) Å	µ = 0.23 mm^−^^1^
*c* = 11.1827 (7) Å	*T* = 293 (2) K
β = 123.342 (4)º	Plate, yellow
*V* = 1508.41 (16) Å^3^	0.60 × 0.47 × 0.23 mm
*Z* = 4	

### Data collection

**Table:**

Stoe IPDS2 diffractometer	3086 independent reflections
Monochromator: plane graphite	2361 reflections with *I* > 2σ(*I*)
Detector resolution: 6.67 pixels mm^-1^	*R*_int_ = 0.078
*T* = 293(2) K	θ_max_ = 26.5º
ω scans	θ_min_ = 1.9º
Absorption correction: integration(*X-RED32*; Stoe & Cie, 2002)	*h* = −9→9
*T*_min_ = 0.876, *T*_max_ = 0.950	*k* = −23→26
8036 measured reflections	*l* = −14→13

### Refinement

**Table:**

Refinement on *F*^2^	Hydrogen site location: inferred from neighbouring sites
Least-squares matrix: full	H-atom parameters constrained
*R*[*F*^2^ > 2σ(*F*^2^)] = 0.045	*w* = 1/[σ^2^(*F*_o_^2^) + (0.072*P*)^2^] where *P* = (*F*_o_^2^ + 2*F*_c_^2^)/3
*wR*(*F*^2^) = 0.121	(Δ/σ)_max_ < 0.001
*S* = 0.99	Δρ_max_ = 0.34 e Å^−^^3^
3086 reflections	Δρ_min_ = −0.40 e Å^−^^3^
200 parameters	Extinction correction: SHELXL97 (Sheldrick, 2008), Fc^*^=KFc[1 + 0.001×Fc^2^λ^3^/sin(2θ)]^-1/4^
Primary atom site location: structure-invariant direct methods	Extinction coefficient: 0.021 (3)
Secondary atom site location: difference Fourier map	

### Special details

**Table:**

Geometry. Bond distances, angles *etc*. have been calculated using the rounded fractional coordinates. All su's are estimated from the variances of the (full) variance-covariance matrix. The cell e.s.d.'s are taken into account in the estimation of distances, angles and torsion angles
Refinement. Refinement on *F*^2^ for ALL reflections except those flagged by the user for potential systematic errors. Weighted *R*-factors *wR* and all goodnesses of fit *S* are based on *F*^2^, conventional *R*-factors *R* are based on *F*, with *F* set to zero for negative *F*^2^. The observed criterion of *F*^2^ > σ(*F*^2^) is used only for calculating -*R*-factor-obs *etc*. and is not relevant to the choice of reflections for refinement. *R*-factors based on *F*^2^ are statistically about twice as large as those based on *F*, and *R*-factors based on ALL data will be even larger.

### Fractional atomic coordinates and isotropic or equivalent isotropic displacement parameters (Å^2^)

**Table:**

	*x*	*y*	*z*	*U*_iso_*/*U*_eq_	
S1	0.38087 (8)	0.27490 (2)	0.37886 (5)	0.0508 (2)	
O1	0.7763 (2)	0.43178 (7)	0.81861 (15)	0.0586 (5)	
O2	0.8121 (3)	0.28677 (7)	0.86144 (15)	0.0639 (5)	
O3	0.8298 (3)	0.18228 (7)	0.85862 (16)	0.0662 (5)	
N1	0.6317 (2)	0.34975 (7)	0.61166 (15)	0.0436 (4)	
C1	0.7315 (3)	0.45323 (8)	0.59125 (18)	0.0411 (5)	
C2	0.7924 (3)	0.47139 (9)	0.7303 (2)	0.0450 (5)	
C3	0.8752 (3)	0.53085 (10)	0.7805 (2)	0.0540 (7)	
C4	0.9000 (3)	0.57192 (9)	0.6957 (3)	0.0576 (7)	
C5	0.8421 (3)	0.55480 (10)	0.5586 (2)	0.0577 (7)	
C6	0.7586 (3)	0.49630 (9)	0.5079 (2)	0.0515 (6)	
C7	0.6487 (3)	0.39169 (8)	0.53568 (19)	0.0442 (5)	
C8	0.5536 (3)	0.29018 (8)	0.55994 (19)	0.0425 (5)	
C9	0.6016 (3)	0.23555 (8)	0.63921 (19)	0.0416 (5)	
C10	0.7575 (3)	0.23103 (9)	0.7957 (2)	0.0452 (6)	
C11	0.9685 (4)	0.28619 (13)	1.0126 (2)	0.0705 (8)	
C12	0.4984 (3)	0.18131 (8)	0.55199 (19)	0.0438 (5)	
C13	0.5079 (3)	0.11475 (9)	0.6026 (2)	0.0549 (6)	
C14	0.3347 (3)	0.07392 (9)	0.4836 (2)	0.0572 (7)	
C15	0.3173 (3)	0.08294 (10)	0.3430 (2)	0.0588 (7)	
C16	0.2502 (4)	0.14989 (10)	0.2891 (2)	0.0593 (7)	
C17	0.3767 (3)	0.19544 (9)	0.41006 (19)	0.0457 (6)	
H1	0.72540	0.39820	0.77740	0.0880*	
H3	0.91420	0.54310	0.87180	0.0650*	
H4	0.95640	0.61170	0.73060	0.0690*	
H5	0.85980	0.58280	0.50200	0.0690*	
H6	0.71910	0.48490	0.41600	0.0620*	
H7	0.60630	0.38190	0.44240	0.0530*	
H11A	1.09630	0.26820	1.02990	0.0850*	
H11B	0.91900	0.26140	1.06020	0.0850*	
H11C	0.99470	0.32850	1.04870	0.0850*	
H13A	0.49300	0.11530	0.68330	0.0660*	
H13B	0.64390	0.09660	0.63460	0.0660*	
H14A	0.36400	0.03000	0.51150	0.0690*	
H14B	0.20130	0.08450	0.47070	0.0690*	
H15A	0.21520	0.05350	0.27260	0.0700*	
H15B	0.45220	0.07430	0.35630	0.0700*	
H16A	0.27210	0.15830	0.21290	0.0710*	
H16B	0.10170	0.15510	0.25090	0.0710*	

### Atomic displacement parameters (Å^2^)

**Table:**

	*U*^11^	*U*^22^	*U*^33^	*U*^12^	*U*^13^	*U*^23^
S1	0.0565 (3)	0.0466 (3)	0.0366 (3)	−0.0026 (2)	0.0176 (2)	0.0017 (2)
O1	0.0796 (9)	0.0573 (8)	0.0470 (8)	−0.0121 (7)	0.0400 (7)	−0.0047 (7)
O2	0.0802 (10)	0.0530 (8)	0.0349 (7)	−0.0107 (7)	0.0166 (7)	0.0015 (6)
O3	0.0735 (9)	0.0563 (9)	0.0482 (8)	0.0110 (7)	0.0204 (7)	0.0089 (7)
N1	0.0454 (7)	0.0411 (8)	0.0389 (8)	0.0005 (6)	0.0197 (6)	0.0023 (6)
C1	0.0400 (8)	0.0424 (9)	0.0416 (9)	0.0054 (7)	0.0228 (7)	0.0039 (7)
C2	0.0482 (9)	0.0446 (9)	0.0475 (10)	0.0020 (7)	0.0297 (8)	0.0005 (8)
C3	0.0593 (11)	0.0515 (11)	0.0558 (12)	−0.0004 (9)	0.0345 (10)	−0.0085 (9)
C4	0.0577 (11)	0.0404 (10)	0.0765 (15)	−0.0011 (8)	0.0381 (11)	−0.0031 (10)
C5	0.0647 (12)	0.0488 (11)	0.0647 (13)	0.0010 (9)	0.0388 (11)	0.0115 (10)
C6	0.0574 (10)	0.0532 (11)	0.0470 (10)	0.0011 (9)	0.0306 (9)	0.0049 (9)
C7	0.0461 (9)	0.0475 (10)	0.0373 (9)	0.0035 (7)	0.0219 (7)	0.0011 (8)
C8	0.0427 (8)	0.0432 (9)	0.0385 (9)	0.0007 (7)	0.0204 (7)	−0.0008 (8)
C9	0.0423 (8)	0.0429 (9)	0.0384 (9)	0.0001 (7)	0.0215 (7)	0.0026 (7)
C10	0.0448 (9)	0.0482 (10)	0.0414 (10)	−0.0014 (7)	0.0230 (8)	0.0022 (8)
C11	0.0794 (15)	0.0785 (15)	0.0361 (10)	−0.0213 (12)	0.0207 (10)	−0.0009 (10)
C12	0.0417 (8)	0.0435 (9)	0.0443 (9)	−0.0005 (7)	0.0225 (8)	0.0007 (8)
C13	0.0586 (11)	0.0464 (10)	0.0527 (11)	−0.0021 (8)	0.0262 (9)	0.0054 (9)
C14	0.0635 (11)	0.0457 (10)	0.0665 (13)	−0.0071 (9)	0.0384 (11)	−0.0037 (10)
C15	0.0636 (11)	0.0529 (11)	0.0619 (13)	−0.0089 (9)	0.0358 (10)	−0.0117 (10)
C16	0.0657 (12)	0.0575 (12)	0.0460 (11)	−0.0116 (9)	0.0251 (10)	−0.0100 (9)
C17	0.0474 (9)	0.0446 (10)	0.0423 (10)	−0.0034 (7)	0.0229 (8)	−0.0017 (8)

### Geometric parameters (Å, °)

**Table:**

S1—C8	1.7342 (18)	C13—C14	1.527 (3)
S1—C17	1.725 (2)	C14—C15	1.516 (3)
O1—C2	1.353 (3)	C15—C16	1.518 (3)
O2—C10	1.333 (2)	C16—C17	1.503 (3)
O2—C11	1.436 (2)	C3—H3	0.9300
O3—C10	1.201 (2)	C4—H4	0.9300
O1—H1	0.8200	C5—H5	0.9300
N1—C8	1.381 (2)	C6—H6	0.9300
N1—C7	1.285 (2)	C7—H7	0.9300
C1—C2	1.410 (3)	C11—H11A	0.9600
C1—C7	1.434 (2)	C11—H11B	0.9600
C1—C6	1.399 (3)	C11—H11C	0.9600
C2—C3	1.384 (3)	C13—H13A	0.9700
C3—C4	1.375 (3)	C13—H13B	0.9700
C4—C5	1.391 (3)	C14—H14A	0.9700
C5—C6	1.367 (3)	C14—H14B	0.9700
C8—C9	1.381 (2)	C15—H15A	0.9700
C9—C10	1.482 (3)	C15—H15B	0.9700
C9—C12	1.432 (2)	C16—H16A	0.9700
C12—C17	1.361 (3)	C16—H16B	0.9700
C12—C13	1.508 (3)		
			
S1···C11^i^	3.543 (2)	C7···H16B^v^	3.0900
S1···C10^ii^	3.468 (3)	C8···H11B^ii^	2.9900
S1···H7	2.6900	C8···H1	3.0600
O1···O2	3.102 (2)	C9···H11B^ii^	2.9900
O1···N1	2.607 (2)	C10···H13A	2.9800
O2···N1	2.694 (2)	C12···H15B	3.0400
O2···O1	3.102 (2)	C17···H14B	2.9600
O3···C4^iii^	3.384 (3)	H1···O2	2.5000
O3···C7^iv^	3.359 (3)	H1···N1	1.8800
O3···C13	2.923 (3)	H1···C7	2.4400
O1···H15B^iv^	2.7200	H1···C8	3.0600
O1···H14B^v^	2.7300	H3···H14B^ix^	2.5200
O2···H1	2.5000	H4···O3^vii^	2.7700
O3···H13A	2.6300	H4···H13B^vii^	2.5700
O3···H13B	2.7700	H5···O3^vii^	2.9000
O3···H11B	2.5800	H5···H16A^x^	2.5900
O3···H4^iii^	2.7700	H6···H7	2.4200
O3···H5^iii^	2.9000	H7···S1	2.6900
O3···H11A	2.6100	H7···H6	2.4200
O3···H7^iv^	2.7200	H7···O3^ii^	2.7200
N1···O1	2.607 (2)	H7···H13A^ii^	2.5300
N1···O2	2.694 (2)	H11A···O3	2.6100
N1···H1	1.8800	H11B···O3	2.5800
C1···C1^vi^	3.552 (3)	H11B···C8^iv^	2.9900
C1···C6^vi^	3.427 (3)	H11B···C9^iv^	2.9900
C2···C6^vi^	3.588 (3)	H13A···O3	2.6300
C4···C7^vi^	3.585 (4)	H13A···C10	2.9800
C4···O3^vii^	3.384 (3)	H13A···H7^iv^	2.5300
C5···C7^vi^	3.468 (4)	H13B···O3	2.7700
C6···C2^vi^	3.588 (3)	H13B···C4^iii^	2.9500
C6···C1^vi^	3.427 (3)	H13B···H4^iii^	2.5700
C7···O3^ii^	3.359 (3)	H14A···H14A^xi^	2.5600
C7···C5^vi^	3.468 (4)	H14B···C17	2.9600
C7···C4^vi^	3.585 (4)	H14B···H3^xii^	2.5200
C10···S1^iv^	3.468 (3)	H14B···O1^xiii^	2.7300
C11···S1^viii^	3.543 (2)	H14B···C2^xiii^	3.0200
C13···O3	2.923 (3)	H15A···C1^xiii^	3.0800
C1···H15A^v^	3.0800	H15A···C2^xiii^	3.0300
C2···H15A^v^	3.0300	H15B···C12	3.0400
C2···H14B^v^	3.0200	H15B···O1^ii^	2.7200
C4···H13B^vii^	2.9500	H16A···H5^xiv^	2.5900
C7···H1	2.4400	H16B···C7^xiii^	3.0900
			
C8—S1—C17	91.89 (9)	C4—C3—H3	120.00
C10—O2—C11	116.56 (17)	C3—C4—H4	119.00
C2—O1—H1	109.00	C5—C4—H4	120.00
C7—N1—C8	122.23 (16)	C4—C5—H5	120.00
C2—C1—C7	121.75 (18)	C6—C5—H5	120.00
C6—C1—C7	119.76 (17)	C1—C6—H6	119.00
C2—C1—C6	118.47 (17)	C5—C6—H6	119.00
O1—C2—C1	121.91 (17)	N1—C7—H7	119.00
O1—C2—C3	118.30 (17)	C1—C7—H7	119.00
C1—C2—C3	119.79 (19)	O2—C11—H11A	109.00
C2—C3—C4	120.16 (19)	O2—C11—H11B	109.00
C3—C4—C5	121.0 (2)	O2—C11—H11C	109.00
C4—C5—C6	119.2 (2)	H11A—C11—H11B	109.00
C1—C6—C5	121.44 (18)	H11A—C11—H11C	110.00
N1—C7—C1	121.58 (17)	H11B—C11—H11C	109.00
S1—C8—N1	122.39 (13)	C12—C13—H13A	109.00
N1—C8—C9	126.74 (16)	C12—C13—H13B	109.00
S1—C8—C9	110.86 (13)	C14—C13—H13A	109.00
C8—C9—C10	124.74 (17)	C14—C13—H13B	109.00
C10—C9—C12	122.39 (16)	H13A—C13—H13B	108.00
C8—C9—C12	112.63 (16)	C13—C14—H14A	109.00
O2—C10—C9	113.48 (16)	C13—C14—H14B	109.00
O3—C10—C9	124.00 (17)	C15—C14—H14A	109.00
O2—C10—O3	122.52 (18)	C15—C14—H14B	109.00
C9—C12—C17	112.61 (16)	H14A—C14—H14B	108.00
C13—C12—C17	120.37 (16)	C14—C15—H15A	110.00
C9—C12—C13	126.99 (16)	C14—C15—H15B	110.00
C12—C13—C14	111.34 (16)	C16—C15—H15A	110.00
C13—C14—C15	111.96 (19)	C16—C15—H15B	110.00
C14—C15—C16	110.40 (18)	H15A—C15—H15B	108.00
C15—C16—C17	109.58 (16)	C15—C16—H16A	110.00
S1—C17—C16	121.34 (14)	C15—C16—H16B	110.00
C12—C17—C16	126.65 (17)	C17—C16—H16A	110.00
S1—C17—C12	112.01 (14)	C17—C16—H16B	110.00
C2—C3—H3	120.00	H16A—C16—H16B	108.00
			
C17—S1—C8—C9	−0.8 (2)	N1—C8—C9—C10	−3.6 (4)
C17—S1—C8—N1	177.8 (2)	S1—C8—C9—C10	174.9 (2)
C8—S1—C17—C12	1.0 (2)	N1—C8—C9—C12	−178.1 (2)
C8—S1—C17—C16	−178.8 (2)	C8—C9—C12—C13	−177.4 (2)
C11—O2—C10—C9	−177.8 (2)	C8—C9—C12—C17	0.4 (3)
C11—O2—C10—O3	1.9 (4)	C12—C9—C10—O3	10.0 (4)
C7—N1—C8—C9	152.2 (3)	C8—C9—C10—O2	15.7 (4)
C7—N1—C8—S1	−26.2 (3)	C8—C9—C10—O3	−164.0 (3)
C8—N1—C7—C1	−179.6 (2)	C12—C9—C10—O2	−170.3 (2)
C2—C1—C6—C5	0.1 (4)	C10—C9—C12—C13	7.9 (4)
C7—C1—C2—O1	0.1 (4)	C10—C9—C12—C17	−174.3 (2)
C6—C1—C7—N1	176.4 (2)	C9—C12—C13—C14	164.9 (2)
C7—C1—C2—C3	178.8 (2)	C9—C12—C17—C16	178.9 (3)
C2—C1—C7—N1	−1.9 (4)	C17—C12—C13—C14	−12.7 (3)
C6—C1—C2—O1	−178.2 (2)	C9—C12—C17—S1	−1.0 (3)
C7—C1—C6—C5	−178.3 (2)	C13—C12—C17—C16	−3.2 (4)
C6—C1—C2—C3	0.5 (4)	C13—C12—C17—S1	177.03 (19)
C1—C2—C3—C4	−0.6 (4)	C12—C13—C14—C15	45.8 (3)
O1—C2—C3—C4	178.1 (2)	C13—C14—C15—C16	−64.5 (3)
C2—C3—C4—C5	0.3 (4)	C14—C15—C16—C17	45.7 (3)
C3—C4—C5—C6	0.3 (4)	C15—C16—C17—C12	−13.8 (4)
C4—C5—C6—C1	−0.4 (4)	C15—C16—C17—S1	166.03 (19)
S1—C8—C9—C12	0.4 (3)		

Symmetry codes: (i) *x*−1, *y*, *z*−1; (ii) *x*, −*y*+1/2, *z*−1/2; (iii) −*x*+2, *y*−1/2, −*z*+3/2; (iv) *x*, −*y*+1/2, *z*+1/2; (v) *x*+1, −*y*+1/2, *z*+1/2; (vi) −*x*+1, −*y*+1, −*z*+1; (vii) −*x*+2, *y*+1/2, −*z*+3/2; (viii) *x*+1, *y*, *z*+1; (ix) −*x*+1, *y*+1/2, −*z*+3/2; (x) −*x*+1, *y*+1/2, −*z*+1/2; (xi) −*x*+1, −*y*, −*z*+1; (xii) −*x*+1, *y*−1/2, −*z*+3/2; (xiii) *x*−1, −*y*+1/2, *z*−1/2; (xiv) −*x*+1, *y*−1/2, −*z*+1/2.

### Hydrogen-bond geometry (Å, °)

**Table:**

*D*—H···*A*	*D*—H	H···*A*	*D*···*A*	*D*—H···*A*
O1—H1···O2	0.82	2.50	3.102 (2)	132
O1—H1···N1	0.82	1.88	2.607 (2)	146
C7—H7···S1	0.93	2.69	3.0725 (19)	105
C15—H15A···Cg^xiii^	0.97	2.92	3.782 (3)	150

Symmetry codes: (xiii) *x*−1, −*y*+1/2, *z*−1/2.
